# Accelerating an Ordered-Subset Low-Dose X-Ray Cone Beam Computed Tomography Image Reconstruction with a Power Factor and Total Variation Minimization

**DOI:** 10.1371/journal.pone.0153421

**Published:** 2016-04-13

**Authors:** Hsuan-Ming Huang, Ing-Tsung Hsiao

**Affiliations:** 1 Medical Physics Research Center, Institute of Radiological Research, Chang Gung University and Chang Gung Memorial Hospital, Taoyuan City, Taiwan; 2 Department of Nuclear Medicine and Neuroscience Research Center, Chang Gung Memorial Hospital, Taoyuan City, Taiwan; 3 Department of Medical Imaging and Radiological Sciences and Healthy Aging Research Center, College of Medicine, Chang Gung University, Taoyuan City, Taiwan; The University of Chicago, UNITED STATES

## Abstract

In recent years, there has been increased interest in low-dose X-ray cone beam computed tomography (CBCT) in many fields, including dentistry, guided radiotherapy and small animal imaging. Despite reducing the radiation dose, low-dose CBCT has not gained widespread acceptance in routine clinical practice. In addition to performing more evaluation studies, developing a fast and high-quality reconstruction algorithm is required. In this work, we propose an iterative reconstruction method that accelerates ordered-subsets (OS) reconstruction using a power factor. Furthermore, we combine it with the total-variation (TV) minimization method. Both simulation and phantom studies were conducted to evaluate the performance of the proposed method. Results show that the proposed method can accelerate conventional OS methods, greatly increase the convergence speed in early iterations. Moreover, applying the TV minimization to the power acceleration scheme can further improve the image quality while preserving the fast convergence rate.

## Introduction

Due to radiation-induced cancer risks and biological perturbations, the use of low-dose X-ray cone beam computed tomography (CBCT) has been gradually gaining attention in many fields including dentistry [1,2], breast imaging [3,4], image-guided radiation therapy [5,6], small animal imaging [7,8] and phase-contrast imaging [9,10]. In general, the low-dose CBCT data acquisition can be achieved by decreasing the milliampere seconds (mAs) per projection view or acquiring a small number of projection data (i.e. sparse views) per rotation [11,12]. However, these dose reduction strategies lead to degradation of image quality, which may directly affect diagnostic accuracy. This problem induced by low dose has made it necessary to have accurate reconstruction algorithms, instead of commonly used analytic reconstruction algorithms such as filtered back-projection.

To improve the quality of low-dose CBCT image, many approaches have been proposed in the past two decades. Typically, iterative reconstruction (IR) methods were proposed to improve spatial resolution and to reduce noise and other artifacts in low-mAs CT [13–17]. In contrast, total-variation (TV) minimization methods were primarily used to suppress streak artifacts and noise in sparse-view CBCT [18–22]. However, both high computational load and slow convergence make them impractical for routine use. Thanks to recent advances in graphics processing unit (GPU) technology, the reconstruction time with IR methods can be reduced dramatically [23–25]. In addition to GPU computing, using ordered subsets (OS) of projection data is a common way to improve the convergence rate [26–30]. Despite the OS acceleration, the number of iterations required to achieve satisfactory image quality is high. Recent studies showed that combing OS-type IR methods with other techniques such as spatially nonuniform optimization transfer [31] and Nesterov’s momentum [32] could improve the initial convergence speed [33,34]. However, these techniques require a couple of relaxation parameters. Tuning the relaxation parameters is inconvenient and tedious since optimizing parameters for ensuring a faster convergence rate remains a challenging issue [33].

In addition to the above-mentioned accelerating techniques, one simple way of accelerating OS-type IR methods is to use a bigger step size or power factor *h*, which has been used in emission tomography [35–37]. Based on the results obtained from previous studies [35–37], the accelerated OS-type algorithm was three or even four times faster than the conventional OS-type algorithm. Therefore, we studied the feasibility of using a power factor to accelerate OS-type IR methods in CT. Moreover, we propose to combine the power acceleration scheme with the TV minimization method to provide better image quality and more acceleration. In this paper, we evaluate the performance of the proposed algorithm using simulation and phantom data. Specifically, we focus on low-dose CBCT image reconstruction.

The remaining part of this paper is organized as follows. In section Method, we describe the power acceleration scheme and the TV minimization in brief, and then describes the combination of an OS-type IR method with the power acceleration scheme and the TV minimization in detail. In the following section, we show results obtained from simulation and measured phantom data. Discussion and conclusion are given in final section.

## Methods

### Acceleration of OS-Type IR Methods Using a Power Factor h

Here, we briefly introduce the concept of accelerating OS-type IR methods using a power factor *h*. Based on the power acceleration scheme [35–37], the OS-type maximum-likelihood expectation-maximization (OSEM) algorithm in emission tomography using a power factor *h* can be expressed in two steps as:

$$f_{j}^{k,l+1}=f_{j}^{k,l}{\left(\frac{1}{\sum_{i\in S_{l}}H_{ij}}\underset{i\in S_{l}}{\sum }\frac{H_{ij}g_{i}}{\sum_{j^{\prime }}H_{ij^{\prime }}f_{j^{\prime }}^{k,l}}\right)}^{h}$$ (1)

$${\hat{f}}_{j}^{k,l+1}=f_{j}^{k,l+1}\frac{\sum_{i\in S_{l}}g_{i}}{\sum_{i\in S_{l}}\sum_{j}H_{ij}f_{j}^{k,l+1}}$$ (2)

where *H*_*ij*_ is the system matrix indicating the probability of photon emitted from voxel *j (j = 1*,*…*,*J)* and detected by detector bin *i (i = 1*,*…*,*I)*, *g*_*i*_ is the projection data at detector bin *i*, *S*_*l*_ denotes the *l*^*th*^ subset projection data, $f_{j}^{k,l}$ is the estimated object activity at voxel *j* and at the *k*^*th*^ iteration and *l*^*th*^ subiteration *(l = 1*,*…*,*L)* and *L* is the number of subsets. Note that there are *L* subiterations for each iteration *k*. The first step is the update equation of OSEM with a power factor *h*. The second step is a multiplication of a rescaling factor to the estimated object activity $f_{j}^{k,l+1}$ at next subiteration *l+1*. The purpose of rescaling is to preserve the total counts in the reconstruction. According to Hwang and Zeng [36], Eq (1) can be rewritten as an additive form and approximated using the Taylor series expansion:

$$f_{j}^{k,l+1}=f_{j}^{k,l}+h\frac{f_{j}^{k,l}}{\sum_{i\in S_{l}}H_{ij}}\left[\underset{i\in S_{l}}{\sum }\frac{H_{ij}}{\sum_{j^{\prime }}H_{ij^{\prime }}f_{j^{\prime }}^{k,l}}\left(g_{i}-\sum_{j^{\prime }}H_{ij^{\prime }}f_{j^{\prime }}^{k,l}\right)\right]\;$$ (3)

This indicates that the power-based accelerated algorithm is almost the same as the original algorithm with a fixed step size. More details can be found in [36].

In a similar manner, we can apply the power acceleration scheme to accelerate the ordered subsets transmission (OSTR) algorithm proposed by Erdoğan and Fessler [27]. The accelerated OSTR (AOSTR) algorithm with a power of *h* can be written as the following two equations (i.e. updating and rescaling steps):

$$\mu_{j}^{k,l+1}=\mu_{j}^{k,l}{\left[1+\frac{L}{\mu_{j}^{k,l}\sum_{i}H_{ij}r_{i}{\bar{b}}_{i}}\underset{i\in S_{l}}{\sum }H_{ij}\left(b_{i}e^{-\underset{j}{\sum }H_{ij}\mu_{j}^{k,l}}-{\bar{b}}_{i}\right)\right]}^{h}$$ (4)

$${\hat{\mu }}_{j}^{k,l+1}=\mu_{j}^{k,l+1}\frac{\sum_{i\in S_{l}}log\left(b_{i}/{\bar{b}}_{i}\right)}{\sum_{i\in S_{l}}\sum_{j}H_{ij}\mu_{j}^{k,l+1}}$$ (5)

where ${\bar{b}}_{i}$ is the observed CT projection data at detector bin *i*, *b*_*i*_ is the blank scan with detector bin *i*, *r*_*i*_ = ∑_*j*_ *H*_*ij*_ and $\mu_{j}^{k,l}$ is the estimated attenuation coefficient at voxel *j* and at the *k*^*th*^ iteration and *l*^*th*^ subiteration. Using the Taylor series expansion, the updating equation in Eq (4) can be simply approximated as:

$$\mu_{j}^{k,l+1}=\mu_{j}^{k,l}+h\left[\frac{L}{\sum_{i}H_{ij}r_{i}{\bar{b}}_{i}}\underset{i\in S_{l}}{\sum }H_{ij}\left(b_{i}e^{-\underset{j}{\sum }H_{ij}\mu_{j}^{k,l}}-{\bar{b}}_{i}\right)\right]$$ (6)

Note that the AOSTR algorithm is the same as the OSTR algorithm with a fixed step size, but the rescaling step in Eq (5) is required in order to preserve the total counts in the reconstruction [35–37]. Based on previous studies [35–37], we expect that the present AOSTR algorithm will provide an appreciable improvement in convergence speed. However, due to the ill-posed reconstruction problem caused by incomplete data [18], noise and artifacts in the image reconstructed using the power acceleration scheme are more prominent after a few iterations. Therefore, we further propose to combine the power acceleration scheme with the TV minimization to provide better image quality and more acceleration.

### Combination of Power Acceleration and TV Minimization

As mentioned previously, many different TV methods have been used to solve various CT problems including bad detector bins, spare-view data and low dose data [18–22]. Basically, the idea of the TV method is to solve the following objective function [18]:

$$\underset{\mu \geq o}{min}||\vec{\mu }|{|}_{TV}\;s.t.\;log\left(b/\bar{b}\right)=H\mu$$ (7)

The TV of the to-be-reconstructed image can be defined as:

$$\begin{array}{l} \|\vec{\mu }{\|}_{TV}=\;\sum_{x,y,z}\left|\vec{\nabla }\mu_{x,y,z}\right|\\ =\sum_{x,y,z}\sqrt{{\left(\mu_{x,y,z}-\mu_{x-1,y,z}\right)}^{2}+{\left(\mu_{x,y,z}-\mu_{x,y-1,z}\right)}^{2}+{\left(\mu_{x,y,z}-\mu_{x,y,z-1}\right)}^{2}} \end{array}$$ (8)

where *x*, *y* and *z* denote the three-dimensional location of the voxel *j*. To minimize the constrained problem in Eq (7), a two-step alternative minimization scheme [18–22] could be used. In the first step, an initially estimated image was reconstructed using IR methods. Herein, we used the present AOSTR algorithm to update the reconstructed image. In the second step, a steepest descent search algorithm described in [19] was performed to minimize the TV of the reconstructed image. Therefore, the proposed reconstruction algorithm, called AOSTR-TV, can be summarized as the following pseudo-code:

1: *k*: = 0, *K*_*TV*_ = 10, $\mu_{j}^{0,0}=0.0002$;

2: while *k* ≤ *K* (main iteration)

3:  for *l = 1*,*2*, …,*L* (subiteration loop)

4:   Update $\mu_{j}^{k,l}$ using AOSTR in Eq (6)

5:   Rescale $\mu_{j}^{k,l+1}$ using Eq (5);$\mu_{x,y,z}^{k_{TV}}:=\mu_{j}^{k,l+1}$;

6:   for *k*_*TV*_ = *1*,*2*, …,*K*_*TV*_

7:    Compute the steepest decent direction *d*_*x*,*y*,*z*_;

8:    $\beta :=max\left(\mu_{x,y,z}^{k_{TV}}\right)÷max\left(\left|d_{x,y,z}\right|\right)$;

9:    $\mu_{x,y,z}^{k_{TV}}=\mu_{x,y,z}^{k_{TV}}-\alpha \times \beta \times d_{x,y,z}$;

10:    *α* = 0.997 × *α*;

11:   end for

12:   $\mu_{j}^{k,l+1}:\;=\mu_{x,y,z}^{k_{TV}}$;

13:  end for

14: *k = k+*1;

15: end while

*α* is the step size for the TV gradient descent procedure. According to [19], the steepest descent direction *d*_*x*,*y*,*z*_ can be defined as:

$$\begin{array}{l} d_{x,y,z}=\frac{\partial {\left\|\vec{\mu }\right\|}_{TV}}{\partial \mu_{x,y,z}^{k_{TV}}}\approx \frac{6\mu_{x,y,z}^{k_{TV}}-\mu_{x-1,y,z}^{k_{TV}}-\mu_{x+1,y,z}^{k_{TV}}-\mu_{x,y-1,z}^{k_{TV}}-\mu_{x,y+1,z}^{k_{TV}}-\mu_{x,y,z-1}^{k_{TV}}-\mu_{x,y,z+1}^{k_{TV}}}{\Delta \mu_{x,y,z}}\\ +\frac{\mu_{x,y,z}^{k_{TV}}-\mu_{x-1,y,z}^{k_{TV}}}{\Delta \mu_{x-1,y,z}}+\frac{\mu_{x,y,z}^{k_{TV}}-\mu_{x+1,y,z}^{k_{TV}}}{\Delta \mu_{x+1,y,z}}+\frac{\mu_{x,y,z}^{k_{TV}}-\mu_{x,y-1,z}^{k_{TV}}}{\Delta \mu_{x,y-1,z}}+\frac{\mu_{x,y,z}^{k_{TV}}-\mu_{x,y+1,z}^{k_{TV}}}{\Delta \mu_{x,y+1,z}}\\ +\frac{\mu_{x,y,z}^{k_{TV}}-\mu_{x,y,z-1}^{k_{TV}}}{\Delta \mu_{x,y,z-1}}+\frac{\mu_{x,y,z}^{k_{TV}}-\mu_{x,y,z+1}^{k_{TV}}}{\Delta \mu_{x,y,z+1}} \end{array}$$ (9)

where

$$\Delta \mu_{x,y,z}={\left[{\left(\mu_{x,y,z}^{k_{TV}}-\mu_{x-1,y,z}^{k_{TV}}\right)}^{2}+{\left(\mu_{x,y,z}^{k_{TV}}-\mu_{x+1,y,z}^{k_{TV}}\right)}^{2}+{\left(\mu_{x,y,z}^{k_{TV}}-\mu_{x,y-1,z}^{k_{TV}}\right)}^{2}+{\left(\mu_{x,y,z}^{k_{TV}}\;-\mu_{x,y+1,z}^{k_{TV}}\right)}^{2}+{\left(\mu_{x,y,z}^{k_{TV}}-\mu_{x,y,z-1}^{k_{TV}}\right)}^{2}+{\left(\mu_{x,y,z}^{k_{TV}}-\mu_{x,y,z+1}^{k_{TV}}\right)}^{2}+\varepsilon \right]}^{\frac{1}{2}}$$ (10)

*ε* (= 10^-8^) is a small positive number to prevent the singularity when calculating *d*_*x*,*y*,*z*_. Note that the proposed AOSTR-TV algorithm with power factor *h* = 1 becomes the OSTR-TV algorithm.

### Practical Implementation of AOSTR-TV

Based on our preliminary tests, the propsoed AOSTR-TV algorithm can provide faster convergence than the OSTR algorithm. However, due to the computation of the rescaling factor and the TV minimization step at each subiteration, the AOSTR-TV algorithm described in the above section requires considerably more computation per iteration as compared to the original OSTR algorithm. Here, performing a TV minimization step at each subiteration was called subiteration-level TV minimization. To save the computation time, we perform the TV minimization step at the last subiteration for each iteration of the AOSTR algorithm, meaning that the TV method is performed once at each iteration of the AOSTR algorithm. For simplicity, this implementation was termed as iteration-level TV minimization. Because of this reason, the rescaling step can be combined with the forward projection of the next subiteration [35–37], and one additional computation of the rescaling will be performed at the last subiteration of each iteration of the AOSTR algorithm. Such implementations could make the AOSTR-TV algorithm an efficient approach for CBCT reconstruction. The implementation of the final efficient AOSTR-TV algorithm can be summarized by the following pseudo-code:

1: *k*: = 0, *K*_*TV*_ = 10,$\mu_{j}^{0,0}=0.0002$;

2: while *k* ≤ *K* (main iteration)

3:  for *l = 1*,*2*, …,*L* (subiteration loop)

4:   Update $\mu_{j}^{k,l}$ using AOSTR in Eq (6)

5:   if *l* <L,

6:     Rescale $\mu_{j}^{k,l+1}$ during the forward projection of the next subiteration;

7:   end if

8:  end for

9  Perform the rescaling step for $\mu_{j}^{k,L}$; $\mu_{x,y,z}^{k_{TV}}:=\mu_{j}^{k,L}$;

10:  for *k*_*TV*_ = *1*,*2*, …,*K*_*TV*_

11:   Compute the steepest decent direction *d*_*x*,*y*,*z*_;

12:   $\beta :\;=max\left(\mu_{x,y,z}^{k_{TV}}\right)÷max\left(\left|d_{x,y,z}\right|\right)$;

13:   $\mu_{x,y,z}^{k_{TV}}=\mu_{x,y,z}^{k_{TV}}-\alpha \times \beta \times d_{x,y,z}$;

14:   *α* = 0.997 × *α*;

15:  end for

16:  $\mu_{j}^{k,L}:\;=\mu_{x,y,z}^{k_{TV}}$;

17: *k = k+*1;

18: end while

### Simulation and Phantom Studies

To evaluate the performance of the proposed AOSTR-TV algorithm, we simulated a CBCT geometry, with a source-to-isocenter distance of 100 cm and source-to-detector distance of 153.6 cm. The test image was a modified 3D Shepp-Logan phantom (128×128×128) generated by Matlab function *phantom*. We reconstructed the image from a 192×192×120 (detector columns × detector rows × projection views) low-dose projection data (10k photons per detector bin). Poisson noise was added to the projection data. The voxel size was 0.208 cm and the detector bin size was 0.213 cm. The simulated phantom had attenuation values of 0.528 cm^-1^ (bone), 0.206 cm^-1^ (water), 0 cm^-1^ (air) and 0.309 cm^-1^ (soft tissue) for the ellipse contour, the background ellipse, the two large ellipses and other small ellipses, respectively. To quantify the accuracy and the convergence speed of the reconstructed image, we used the relative root mean square error (RRMSE) defined as:

$$\text{RRMSE}=\sqrt{\frac{\sum_{j}{\left(\mu_{j}^{k,l+1}-\mu_{j}^{true}\right)}^{2}}{\sum_{j}{\left(\mu_{j}^{true}\right)}^{2}}}$$ (11)

where $\mu_{j}^{true}$ is the true attenuation value at voxel *j*. We also compared the proposed AOSTR-TV algorithm to other algorithms including OSTR, AOSTR and OSTR-TV.

The performance of the proposed AOSTR-TV algorithm was also evaluated using the experimental phantom data obtained from [38]. The phantom data was collected from the X-ray Volumetric Imager (XVI, Elekta Oncology Systems, Norcross, GA) with a typical setting of 120 kV, 40 mA, and 40 ms/frame. The Catphan phantom (The Phantom Laboratory, Inc., Salem, NY) was scanned at 669 projection views over 360 degrees. The number of detector bins was 1024×1024 (0.4×0.4 mm). To simulate the low-dose CT data, images with the size of 512×512×512 were reconstructed using 120 projections that were evenly extracted from the total 669 projections.

## Results

### Simulation Study

**Fig 1:**
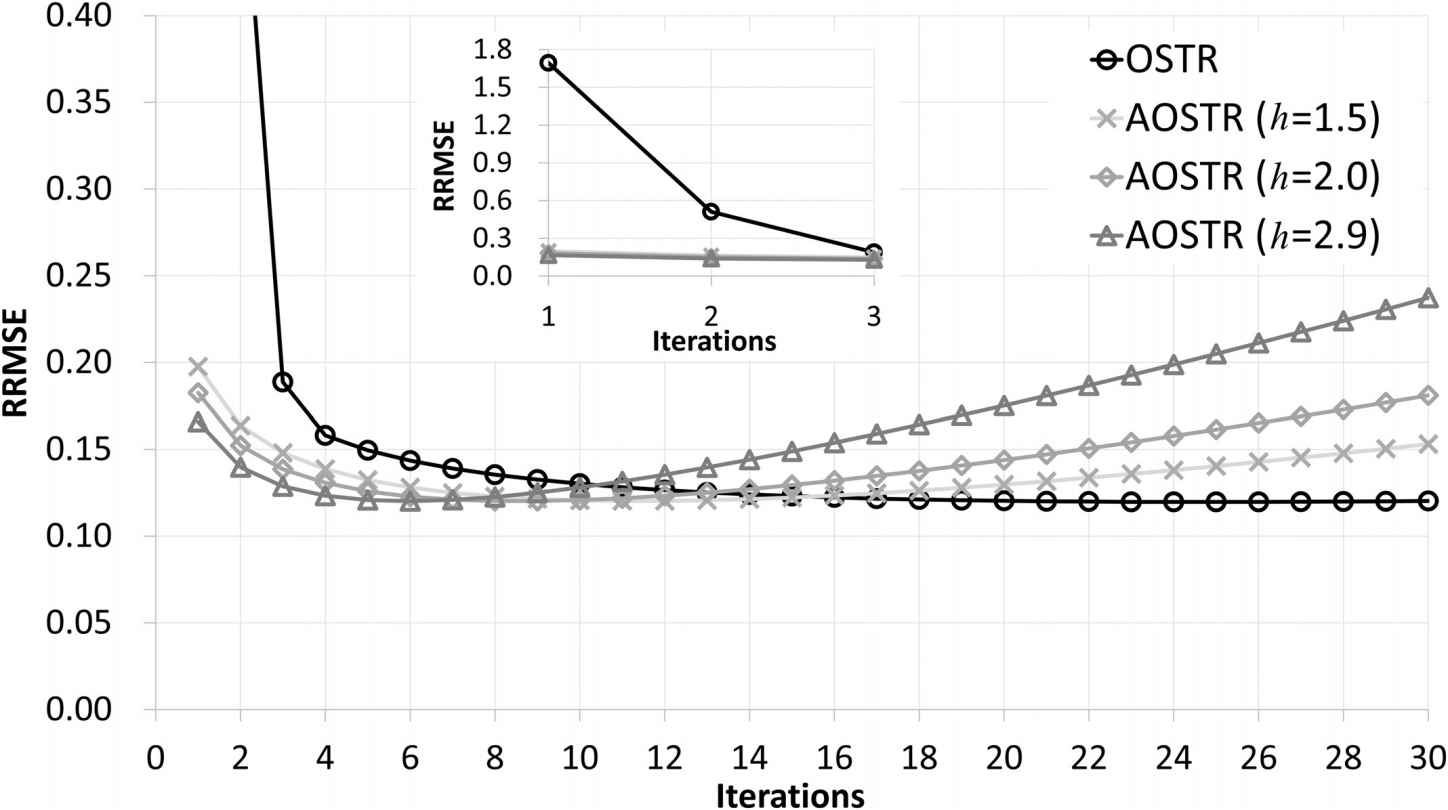
Simulation data: RRMSE values versus iteration numbers (30 subsets) for the OSTR and AOSTR (*h* = 1.5, 2.0 and 2.9) algorithms. A zoomed-out view of early iterations of this figure is also displayed.

**Fig 2:**
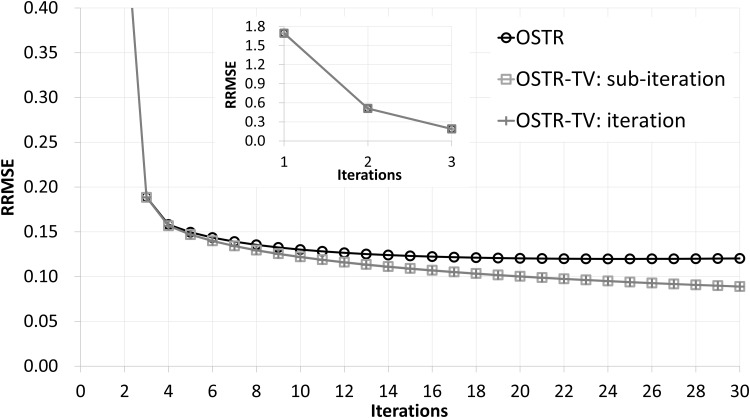
Simulation data: RRMSE values versus iteration numbers (30 subsets) for the OSTR and OSTR-TV algorithms. For OSTR-TV, the TV step was performed at each subiteration (*α* = 0.00003) or at each iteration (*α* = 0.001). A zoomed-out view of early iterations of this figure is also displayed.

Fig 1 illustrates RRMSE values versus iteration numbers (30 subsets) for OSTR and AOSTR (*h* = 1.5, 2.0 and 2.9) algorithms. The simulation results show that the power *h* of the AOSTR algorithm can be up to 2.9. Comparing the results of AOSTR with those of OSTR, it can be seen that the present AOSTR algorithm can provide faster convergence rate since its RRMSE values decrease rapidly. Fig 2 shows RRMSE values versus iteration numbers (30 subsets) for OSTR and OSTR-TV algorithms. Despite using different values of *α*, both the iteration-level OSTR-TV (*α* = 0.001) and the subiteration-level OSTR-TV (*α* = 0.00003) algorithms have almost the same convergence rate. This result indicates that the implementation of the iteration-level AOSTR-TV could be feasible and efficient. Therefore, all of the results of the OSTR-TV and AOSTR-TV algorithms shown below were obtained using the iteration-level TV minimization.

**Fig 3:**
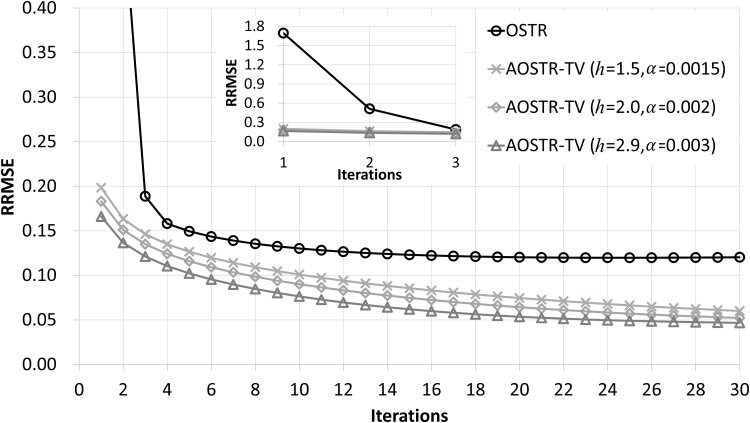
Simulation data: RRMSE values versus iteration numbers (30 subsets) for the OSTR and AOSTR-TV (h = 1.5 with *α* = 0.0015, *h* = 2.0 with *α* = 0.002 and *h* = 2.9 with *α* = 0.003) algorithms. A zoomed-out view of early iterations of this figure is also displayed.

**Fig 4:**
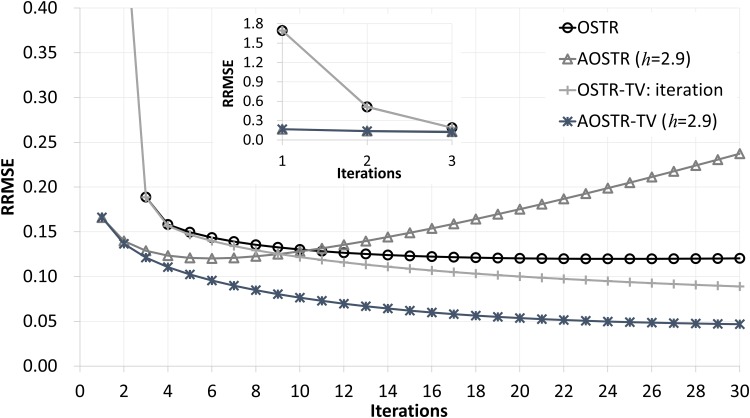
Simulation data: RRMSE values versus iteration numbers (30 subsets) for the OSTR, AOSTR (h = 2.9), OSTR-TV (*α* = 0.001) and AOSTR-TV (h = 2.9 and *α* = 0.003) algorithms. For OSTR-TV and AOSTR-TV, the TV step was performed at each iteration. A zoomed-out view of early iterations of this figure is also displayed.

**Fig 5:**
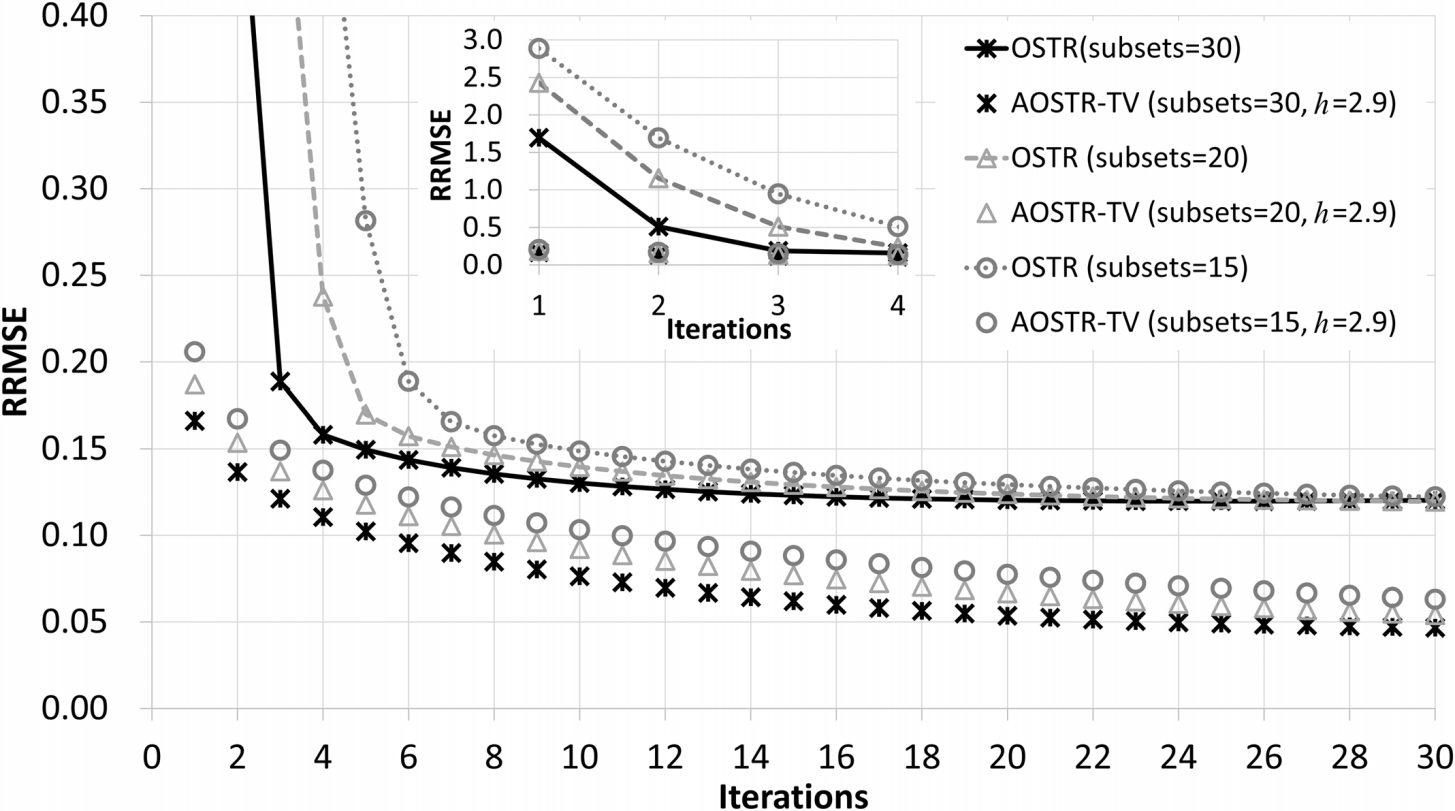
Simulation data: RRMSE values versus iteration numbers for the OSTR and AOSTR-TV algorithms with 15, 20 and 30 subsets. For AOSTR-TV, we used *h* = 2.9 for all subsets and *α* = 0.0015, 0.002 and 0.003 for 15, 20 and 30 subsets, respectively. A zoomed-out view of early iterations of this figure is also displayed.

**Fig 6:**
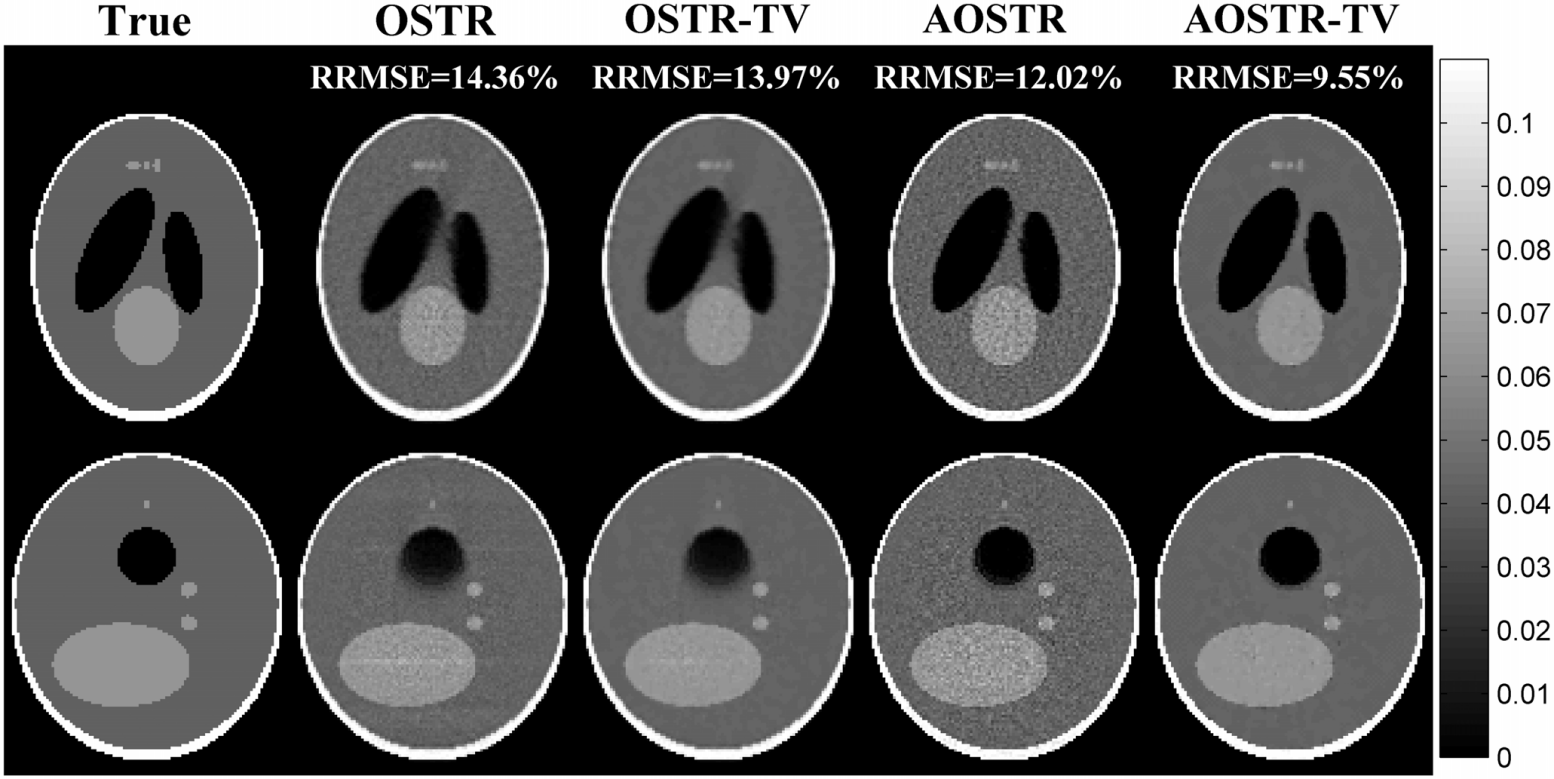
Simulation data: a transaxial view (upper row) and a sagittal view (lower row) of true images and images reconstructed using the OSTR, OSTR-TV (*α* = 0.001), AOSTR (h = 2.9) and AOSTR-TV (h = 2.9 and *α* = 0.003) algorithms. For each reconstructed image, we ran 6 iterations with 30 subsets.

Fig 3 illustrates RRMSE values versus iteration numbers (30 subsets) for OSTR and AOSTR-TV (*h* = 1.5 with *α* = 0.0015, *h* = 2.0 with *α* = 0.002 and *h* = 2.9 with *α* = 0.003) algorithms. As expected, the AOSTR-TV algorithm with a higher power value has a faster convergence rate than that with a lower power value. In Fig 4, the RRMSE values of AOSTR-TV (*h* = 2.9 and *α* = 0.003) versus iteration numbers (30 subsets) were plotted and compared with those of OSTR, AOSTR (*h* = 2.9) and OSTR-TV (*α* = 0.001). It can be easily seen that the present AOSTR-TV algorithm outperforms all other algorithms in terms of RRMSE reduction. Furthermore, as illustrated in Fig 5, the present AOSTR-TV algorithm works well under different numbers of subsets (15, 20 and 30). The RRMSE values of Figs 1–5 were summarized in the supplementary table (S1A–S1E Table). Finally, Fig 6 displays a transaxial view (upper row) and a sagittal view (lower row) of true images and images reconstructed using the OSTR, OSTR-TV (*α* = 0.001), AOSTR (*h* = 2.9) and AOSTR-TV (*h* = 2.9 and *α* = 0.003) algorithms. For each reconstructed image, we ran 6 iterations with 30 subsets. Compared with all other algorithms, the proposed AOSTR-TV algorithm requires les iterations to achieve better image quality.

To compare the computational cost, the reconstruction time was evaluated for both OSTR and AOSTR-TV algorithms. The image reconstruction was performed on a desktop computer equipped with an Intel i7-5960 CPU at 3.0 GHz and 64 GB random-access memory. The execution times were 23.04 and 24.02 seconds per iteration for OSTR and AOSTR-TV, respectively. Calculating the rescaling factor and the iteration-level TV minimization increased the runtime by 4%, but the increase in computation time was minor compared to the acceleration given by the proposed scheme.

### Phantom Study

**Fig 7:**
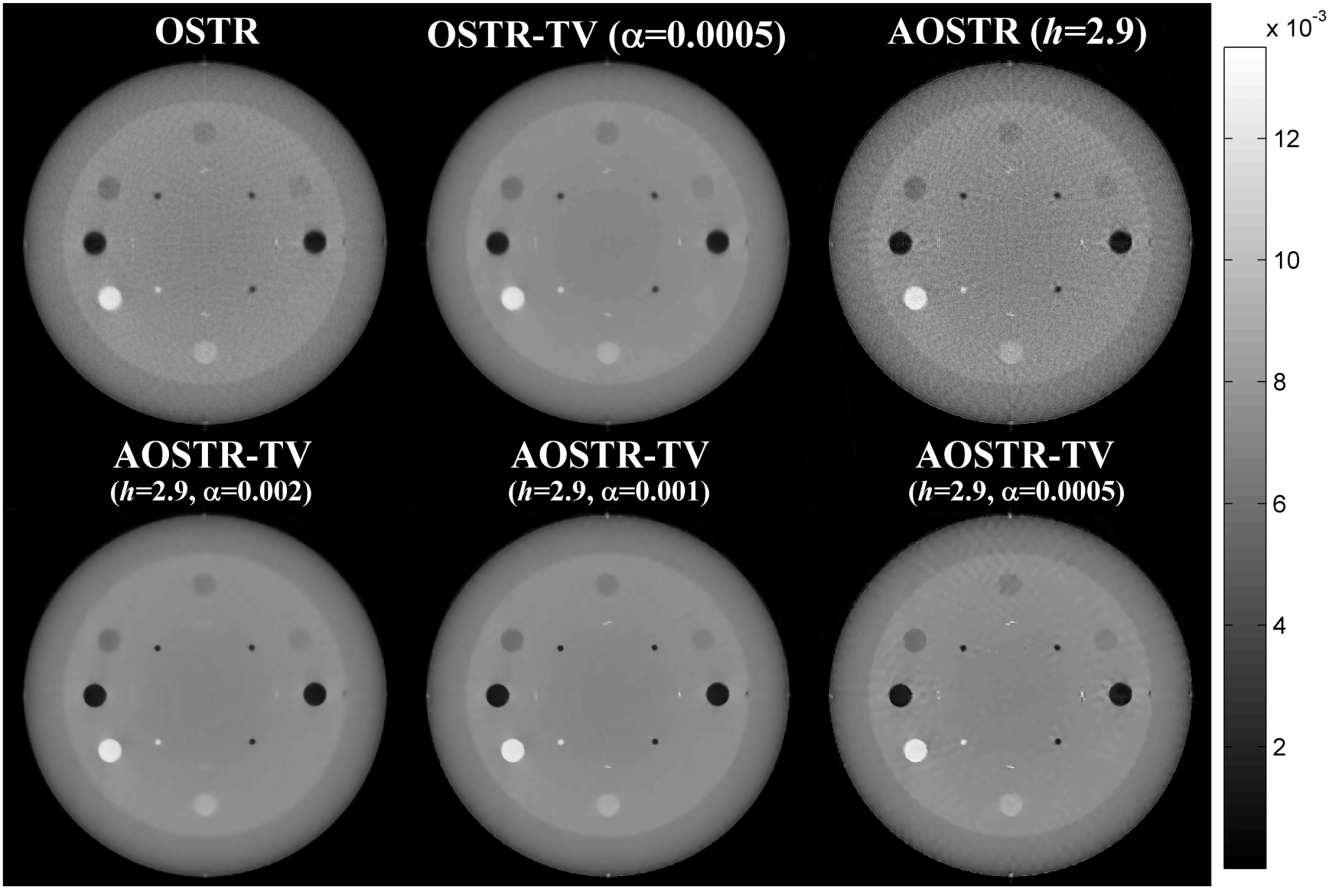
Catphan phantom data (contrast slice): images reconstructed using the OSTR, OSTR-TV (*α* = 0.0005), AOSTR (*h* = 2.9) and AOSTR-TV (*h* = 2.9 with *α* = 0.002, 0.001 and 0.0005) algorithms. For each reconstructed image, we ran 10 iterations with 30 subsets.

**Fig 8:**
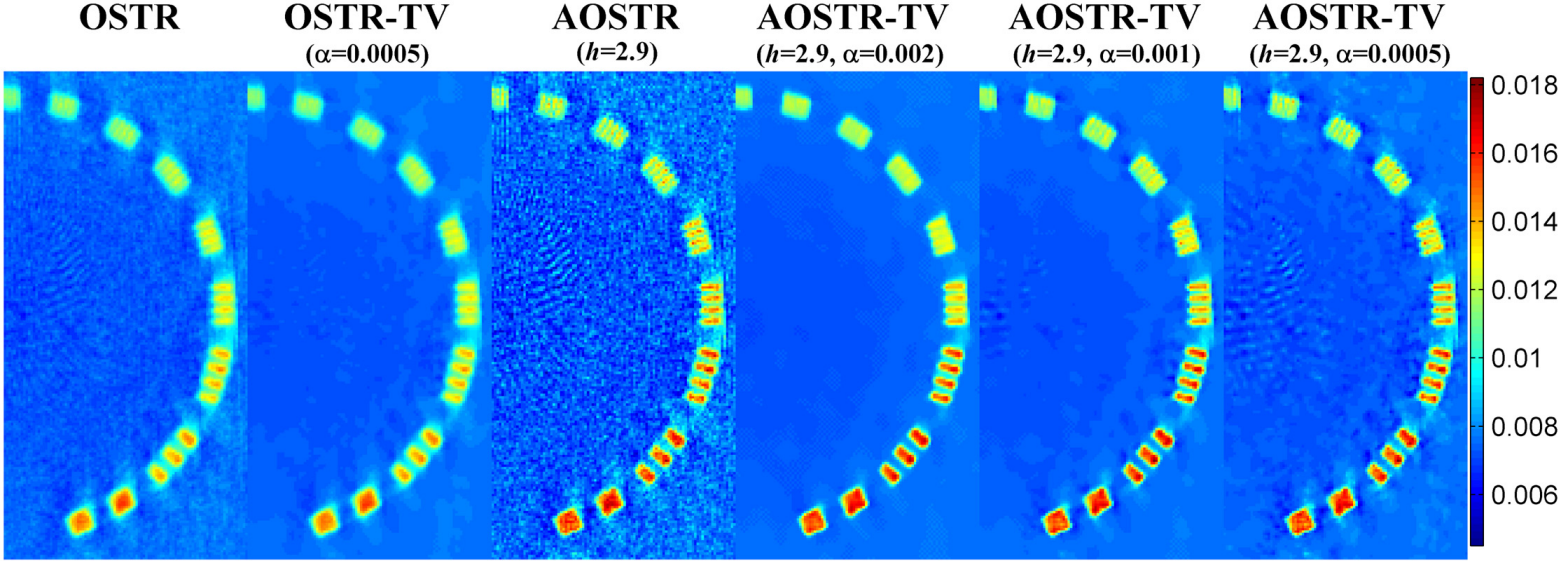
Catphan phantom data (zoomed-in resolution slice): images reconstructed using the OSTR, OSTR-TV (*α* = 0.0005), AOSTR (*h* = 2.9) and AOSTR-TV (*h* = 2.9 with *α* = 0.002, 0.001 and 0.0005) algorithms. For each reconstructed image, we ran 10 iterations with 30 subsets.

**Fig 9:**
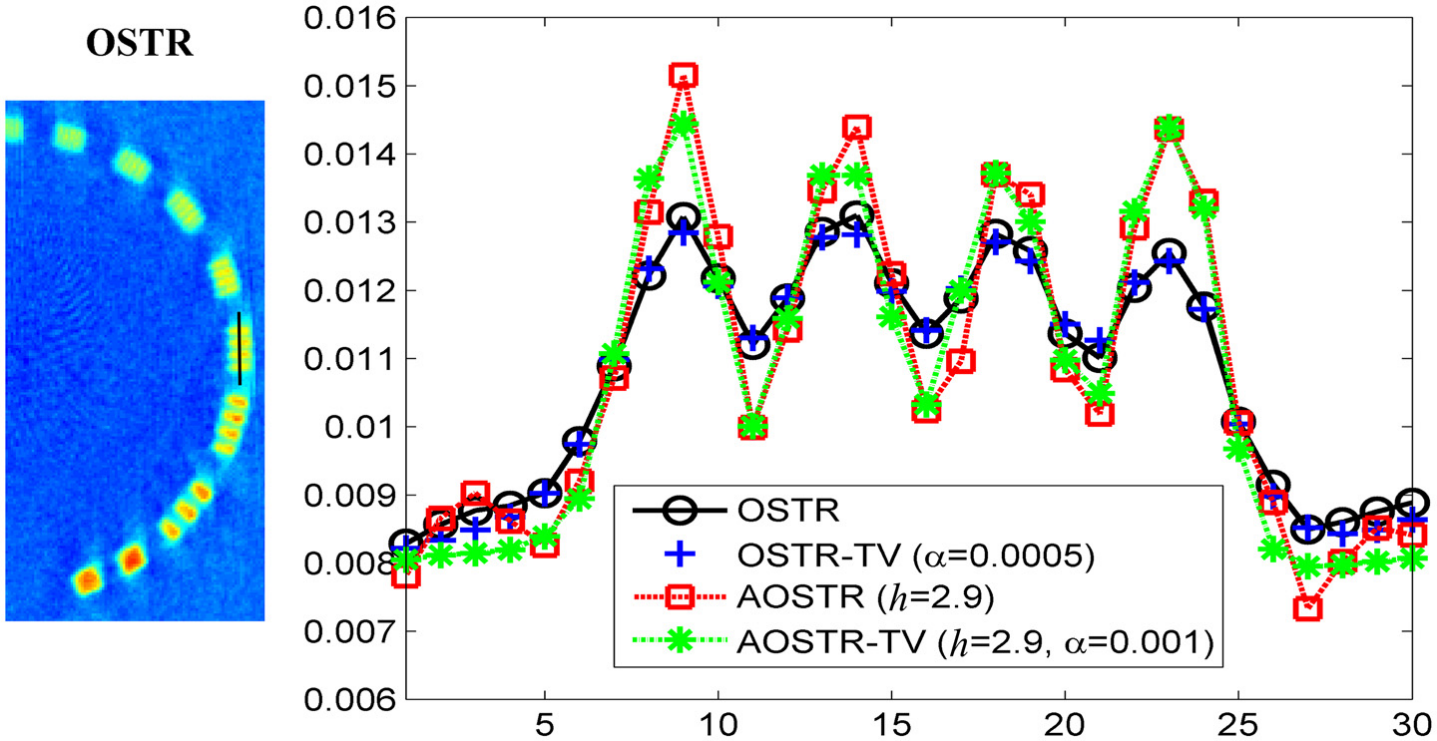
Vertical profiles crossing the four line pairs for OSTR, OSTR-TV (*α* = 0.0005), AOSTR (*h* = 2.9) and AOSTR-TV (*h* = 2.9 and *α* = 0.001) reconstructions shown in Fig 8.

To investigate whether IR algorithm can provide satisfactory image quality after few iterations, all IR algorithms ran for 10 iterations with 30 subsets. Figs 7 and 8 illustrate the contrast slice and the resolution slice (zoomed-in view), respectively, reconstructed using OSTR, OSTR-TV (*α* = 0.0005), AOSTR (*h* = 2.9) and AOSTR-TV (*h* = 2.9 with *α* = 0.002, 0.001 and 0.0005) algorithms. To give a more detailed comparison, the vertical profiles crossing the line pairs are displayed in Fig 9. Compared with the OSTR and OSTR-TV algorithms, both AOSTR and AOSTR-TV algorithms led to an appreciable enhancement in convergence rate. Moreover, the AOSTR-TV algorithm performs better than the AOSTR algorithm in terms of noise reduction. For the AOSTR-TV algorithm, *α* = 0.001 seemed to provide a better compromise between noise suppression and resolution loss.

## Discussion

Due to potential risks associated with CT radiation dose, there has been a tendency to reduce radiation dose in CT [11,12]. However, the image quality degradation caused by noisy and incomplete data is a major obstacle for routinely using low-dose CT. To solve this problem, various methods such as IR [13–17] and TV minimization [18–22] have been proposed. Further improvements including GPU [23–25] and OS [26–30] make them practical for use in low-dose CT. For more routine clinical applications of low-dose CT, developing a faster and more accurate IR algorithm is required.

To address this issue, we presented the AOSTR-TV algorithm which combines the power acceleration scheme [35–37] with the TV minimization method [19] for faster convergence. Results obtained from simulation and phantom data show that the present AOSTR-TV algorithm can rapidly converge to a better solution with a lower RRMSE than other algorithms. Similar to other accelerated algorithms [18–22,31,33], the proposed AOSTR-TV algorithm has a couple of parameters which control the convergence speed of the algorithm. Among these parameters, two parameters are important: the power factor *h* for the acceleration of the OSTR algorithm and the step-size *α* for the TV gradient descent optimization. In this study, the upper limit of the power *h* was 2.9, which was very close to the value (= 3) used in emission tomography [35,36]. This indicates that the upper limit of the power *h* may be relatively insensitive to data types and other factors such as the number of subsets, system’s geometries, image structures and noise levels [36,37]. We also observed that the accelerated algorithm with a power of 2.9 never diverged for all cases in this study. So, the value of *h* = 2.9 appears to be a reasonable choice overall.

For simulation data, we selected *α* values that gave the best performance for AOSTR-TV in terms of RRMSE. For phantom data, we used three different values of *α* (= 0.002, 0.001 and 0.0005). As shown in Figs 7 and 8, increasing values of *α* led to reduction in noise and resolution. A good trade-off between noise suppression and resolution loss can be observed when *α* = 0.001. As for the optimal selection of *α*, it remains an open issue. Some step-size adaption schemes such as a backtracking line search [22,39] and an improved TV constrained reconstruction [40] may provide a good alternative to the selection of *α*. However, such dynamic step-size adaption schemes may not guarantee the best image quality [41]. Moreover, additional computation is required to calculate the step size. As a result, the automatic selection method for the step size may not be fast in convergence in terms of computational time. We leave further optimization of *α* as a future work.

Like the OSTR algorithm and other OS-type IR algorithms [26–30], the AOSTR and AOSTR-TV algorithms do not converge to the minimum of the objective function. However, as shown in Figs 4 and 5, the proposed AOSTR-TV algorithm could rapidly decrease RRMSE in the early iterations and maintain a noticeably lower RRMSE as compared to other algorithms. Also, in practice, one would run few iterations for saving reconstruction time. Because of these reasons, the present AOSTR-TV algorithm should have practical merits. To ensure fast and global convergence, alternatively, one can run few iterations of AOSTR-TV and switch to a convergent algorithm such as the separable paraboloidal surrogates [27] and the transmission incremental optimization transfer algorithm [42].

In this study, we implemented the AOSTR-TV algorithm. Because of the iteration-level TV minimization step and the combination of the rescaling process with the forward projection of the next subiteration, the proposed AOSTR-TV algorithm had a slight increase (~4%) in runtime per iteration compared to the OSTR algorithm. As for the TV minimization method, further improvement will be possible through the combination of the power acceleration scheme [35–37] with other TV techniques including an adaptive sinogram restoration method [21], a conjugate gradient method [22] and an anisotropic method [20]. We leave these possibilities to future extensions.

## Conclusion

We introduced the power acceleration scheme to OS-type low-dose CT reconstruction methods. Furthermore, we proposed to combine the accelerated technique with the TV minimization for low-dose CBCT reconstruction. The performance of the proposed method was evaluated using both simulation and phantom data. Results demonstrate that the proposed method can achieve faster convergence and better image quality than conventional OS-type methods. Such advantages could make the proposed AOSTR-TV algorithm an appealing method for fast and accurate reconstruction of low-dose CBCT data.

## Supporting Information

S1 Table **S1A Table**. RRMSE of Fig 1. **S1B Table**. RRMSE of Fig 2. **S1C Table**. RRMSE of Fig 3. **S1D Table**. RRMSE of Fig 4. **S1E Table**. RRMSE of Fig 5.
(DOCX)
