# Supplementary material for: Accelerating an Ordered-Subset Low-Dose X-Ray Cone Beam Computed Tomography Image Reconstruction with a Power Factor and Total Variation Minimization
Source: PLoS One. 2016 Apr 13;11(4):e0153421. doi: 10.1371/journal.pone.0153421 (PMC4830553; doi:10.1371/journal.pone.0153421)
Supplement: S1 Table — S1A Table. RRMSE of Fig 1. S1B Table. RRMSE of Fig 2. S1C Table. RRMSE of Fig 3. S1D Table. RRMSE of Fig 4. S1E Table. RRMSE of Fig 5. (DOCX) [file pone.0153421.s001.docx]

**S1A Table. RRMSE of Fig. 1**

| Subsets=30 | RRMSE | | | |
| --- | --- | --- | --- | --- |
| Iterations | OSTR | AOSTR  (h=1.5) | AOSTR  (h=2.0) | AOSTR  (h=2.9) |
| 1 | 1.6944 | 0.1976 | 0.1825 | 0.1659 |
| 2 | 0.5108 | 0.1636 | 0.1523 | 0.1398 |
| 3 | 0.1887 | 0.1481 | 0.1386 | 0.1288 |
| 4 | 0.1581 | 0.1387 | 0.1308 | 0.1233 |
| 5 | 0.1495 | 0.1324 | 0.1259 | 0.1208 |
| 6 | 0.1436 | 0.1281 | 0.1228 | 0.1202 |
| 7 | 0.1391 | 0.1250 | 0.1211 | 0.1209 |
| 8 | 0.1355 | 0.1229 | 0.1203 | 0.1226 |
| 9 | 0.1326 | 0.1215 | 0.1203 | 0.1250 |
| 10 | 0.1302 | 0.1206 | 0.1208 | 0.1281 |
| 11 | 0.1282 | 0.1203 | 0.1219 | 0.1316 |
| 12 | 0.1266 | 0.1203 | 0.1233 | 0.1355 |
| 13 | 0.1252 | 0.1207 | 0.1251 | 0.1397 |
| 14 | 0.1241 | 0.1214 | 0.1272 | 0.1442 |
| 15 | 0.1231 | 0.1223 | 0.1295 | 0.1489 |
| 16 | 0.1223 | 0.1234 | 0.1320 | 0.1538 |
| 17 | 0.1217 | 0.1248 | 0.1348 | 0.1589 |
| 18 | 0.1211 | 0.1263 | 0.1377 | 0.1642 |
| 19 | 0.1207 | 0.1279 | 0.1407 | 0.1697 |
| 20 | 0.1204 | 0.1297 | 0.1439 | 0.1752 |
| 21 | 0.1201 | 0.1317 | 0.1472 | 0.1809 |
| 22 | 0.1199 | 0.1337 | 0.1506 | 0.1868 |
| 23 | 0.1198 | 0.1358 | 0.1541 | 0.1927 |
| 24 | 0.1198 | 0.1381 | 0.1577 | 0.1988 |
| 25 | 0.1197 | 0.1404 | 0.1614 | 0.2049 |
| 26 | 0.1198 | 0.1428 | 0.1652 | 0.2112 |
| 27 | 0.1199 | 0.1453 | 0.1690 | 0.2176 |
| 28 | 0.1200 | 0.1478 | 0.1729 | 0.2240 |
| 29 | 0.1201 | 0.1504 | 0.1769 | 0.2306 |
| 30 | 0.1203 | 0.1531 | 0.1810 | 0.2373 |

**S1B Table. RRMSE of Fig. 2**

| Subsets=30 | RRMSE | | |
| --- | --- | --- | --- |
| Iterations | OSTR | OSTR-TV: sub-iteration | OSTR-TV: iteration |
| 1 | 1.6944 | 1.6929 | 1.6934 |
| 2 | 0.5108 | 0.5099 | 0.5102 |
| 3 | 0.1887 | 0.1885 | 0.1885 |
| 4 | 0.1581 | 0.1568 | 0.1566 |
| 5 | 0.1495 | 0.1470 | 0.1468 |
| 6 | 0.1436 | 0.1399 | 0.1397 |
| 7 | 0.1391 | 0.1344 | 0.1341 |
| 8 | 0.1355 | 0.1297 | 0.1293 |
| 9 | 0.1326 | 0.1257 | 0.1253 |
| 10 | 0.1302 | 0.1221 | 0.1218 |
| 11 | 0.1282 | 0.1190 | 0.1186 |
| 12 | 0.1266 | 0.1162 | 0.1158 |
| 13 | 0.1252 | 0.1136 | 0.1133 |
| 14 | 0.1241 | 0.1113 | 0.1109 |
| 15 | 0.1231 | 0.1091 | 0.1088 |
| 16 | 0.1223 | 0.1071 | 0.1068 |
| 17 | 0.1217 | 0.1053 | 0.1049 |
| 18 | 0.1211 | 0.1035 | 0.1032 |
| 19 | 0.1207 | 0.1019 | 0.1016 |
| 20 | 0.1204 | 0.1004 | 0.1001 |
| 21 | 0.1201 | 0.0990 | 0.0987 |
| 22 | 0.1199 | 0.0976 | 0.0973 |
| 23 | 0.1198 | 0.0963 | 0.0961 |
| 24 | 0.1198 | 0.0951 | 0.0949 |
| 25 | 0.1197 | 0.0940 | 0.0938 |
| 26 | 0.1198 | 0.0929 | 0.0927 |
| 27 | 0.1199 | 0.0918 | 0.0917 |
| 28 | 0.1200 | 0.0908 | 0.0907 |
| 29 | 0.1201 | 0.0899 | 0.0898 |
| 30 | 0.1203 | 0.0890 | 0.0889 |

**S1C Table. RRMSE of Fig. 3**

| Subsets=30 | RRMSE | | | |
| --- | --- | --- | --- | --- |
| Iterations | OSTR | AOSTR-TV  (h=1.5,α=0.0015) | AOSTR-TV  (h=2.0,α=0.002) | AOSTR-TV  (h=2.9,α=0.003) |
| 1 | 1.6944 | 0.1984 | 0.1832 | 0.1661 |
| 2 | 0.5108 | 0.1634 | 0.1511 | 0.1365 |
| 3 | 0.1887 | 0.1463 | 0.1350 | 0.1212 |
| 4 | 0.1581 | 0.1350 | 0.1242 | 0.1105 |
| 5 | 0.1495 | 0.1266 | 0.1159 | 0.1023 |
| 6 | 0.1436 | 0.1198 | 0.1092 | 0.0955 |
| 7 | 0.1391 | 0.1141 | 0.1035 | 0.0898 |
| 8 | 0.1355 | 0.1092 | 0.0985 | 0.0848 |
| 9 | 0.1326 | 0.1049 | 0.0941 | 0.0803 |
| 10 | 0.1302 | 0.1009 | 0.0901 | 0.0764 |
| 11 | 0.1282 | 0.0974 | 0.0865 | 0.0729 |
| 12 | 0.1266 | 0.0941 | 0.0832 | 0.0697 |
| 13 | 0.1252 | 0.0910 | 0.0802 | 0.0668 |
| 14 | 0.1241 | 0.0882 | 0.0774 | 0.0643 |
| 15 | 0.1231 | 0.0856 | 0.0748 | 0.0620 |
| 16 | 0.1223 | 0.0831 | 0.0724 | 0.0599 |
| 17 | 0.1217 | 0.0808 | 0.0701 | 0.0580 |
| 18 | 0.1211 | 0.0786 | 0.0680 | 0.0564 |
| 19 | 0.1207 | 0.0766 | 0.0661 | 0.0549 |
| 20 | 0.1204 | 0.0746 | 0.0643 | 0.0536 |
| 21 | 0.1201 | 0.0728 | 0.0626 | 0.0524 |
| 22 | 0.1199 | 0.0711 | 0.0611 | 0.0514 |
| 23 | 0.1198 | 0.0694 | 0.0596 | 0.0505 |
| 24 | 0.1198 | 0.0679 | 0.0583 | 0.0498 |
| 25 | 0.1197 | 0.0664 | 0.0571 | 0.0491 |
| 26 | 0.1198 | 0.0650 | 0.0560 | 0.0485 |
| 27 | 0.1199 | 0.0637 | 0.0549 | 0.0480 |
| 28 | 0.1200 | 0.0624 | 0.0540 | 0.0475 |
| 29 | 0.1201 | 0.0612 | 0.0531 | 0.0471 |
| 30 | 0.1203 | 0.0601 | 0.0523 | 0.0467 |

**S1D Table. RRMSE of Fig. 4**

| Subsets=30 | RRMSE | | | |
| --- | --- | --- | --- | --- |
| Iterations | OSTR | AOSTR  (h=2.9) | OSTR-TV: iteration | AOSTR-TV  (h=2.9) |
| 1 | 1.6944 | 0.1659 | 1.6934 | 0.1661 |
| 2 | 0.5108 | 0.1398 | 0.5102 | 0.1365 |
| 3 | 0.1887 | 0.1288 | 0.1885 | 0.1212 |
| 4 | 0.1581 | 0.1233 | 0.1566 | 0.1105 |
| 5 | 0.1495 | 0.1208 | 0.1468 | 0.1023 |
| 6 | 0.1436 | 0.1202 | 0.1397 | 0.0955 |
| 7 | 0.1391 | 0.1209 | 0.1341 | 0.0898 |
| 8 | 0.1355 | 0.1226 | 0.1293 | 0.0848 |
| 9 | 0.1326 | 0.1250 | 0.1253 | 0.0803 |
| 10 | 0.1302 | 0.1281 | 0.1218 | 0.0764 |
| 11 | 0.1282 | 0.1316 | 0.1186 | 0.0729 |
| 12 | 0.1266 | 0.1355 | 0.1158 | 0.0697 |
| 13 | 0.1252 | 0.1397 | 0.1133 | 0.0668 |
| 14 | 0.1241 | 0.1442 | 0.1109 | 0.0643 |
| 15 | 0.1231 | 0.1489 | 0.1088 | 0.0620 |
| 16 | 0.1223 | 0.1538 | 0.1068 | 0.0599 |
| 17 | 0.1217 | 0.1589 | 0.1049 | 0.0580 |
| 18 | 0.1211 | 0.1642 | 0.1032 | 0.0564 |
| 19 | 0.1207 | 0.1697 | 0.1016 | 0.0549 |
| 20 | 0.1204 | 0.1752 | 0.1001 | 0.0536 |
| 21 | 0.1201 | 0.1809 | 0.0987 | 0.0524 |
| 22 | 0.1199 | 0.1868 | 0.0973 | 0.0514 |
| 23 | 0.1198 | 0.1927 | 0.0961 | 0.0505 |
| 24 | 0.1198 | 0.1988 | 0.0949 | 0.0498 |
| 25 | 0.1197 | 0.2049 | 0.0938 | 0.0491 |
| 26 | 0.1198 | 0.2112 | 0.0927 | 0.0485 |
| 27 | 0.1199 | 0.2176 | 0.0917 | 0.0480 |
| 28 | 0.1200 | 0.2240 | 0.0907 | 0.0475 |
| 29 | 0.1201 | 0.2306 | 0.0898 | 0.0471 |
| 30 | 0.1203 | 0.2373 | 0.0889 | 0.0467 |

**S1E Table. RRMSE of Fig. 5**

|  | RRMSE | | | | | |
| --- | --- | --- | --- | --- | --- | --- |
| Iterations | OSTR  subsets=30 | AOSTR-TV  subsets=30, h=2.9 | OSTR  subsets=20 | AOSTR-TV  subsets=20, h=2.9 | OSTR  subsets=15 | AOSTR-TV  subsets=15, h=2.9 |
| 1 | 1.6944 | 0.1661 | 2.4282 | 0.1871 | 2.8861 | 0.2058 |
| 2 | 0.5108 | 0.1365 | 1.1559 | 0.1535 | 1.6942 | 0.1672 |
| 3 | 0.1887 | 0.1212 | 0.5109 | 0.1371 | 0.9461 | 0.1492 |
| 4 | 0.1581 | 0.1105 | 0.2377 | 0.1261 | 0.5108 | 0.1375 |
| 5 | 0.1495 | 0.1023 | 0.1698 | 0.1178 | 0.2816 | 0.1290 |
| 6 | 0.1436 | 0.0955 | 0.1574 | 0.1111 | 0.1888 | 0.1221 |
| 7 | 0.1391 | 0.0898 | 0.1511 | 0.1054 | 0.1655 | 0.1164 |
| 8 | 0.1355 | 0.0848 | 0.1464 | 0.1005 | 0.1575 | 0.1115 |
| 9 | 0.1326 | 0.0803 | 0.1427 | 0.0961 | 0.1526 | 0.1072 |
| 10 | 0.1302 | 0.0764 | 0.1395 | 0.0922 | 0.1488 | 0.1033 |
| 11 | 0.1282 | 0.0729 | 0.1369 | 0.0886 | 0.1455 | 0.0998 |
| 12 | 0.1266 | 0.0697 | 0.1346 | 0.0854 | 0.1428 | 0.0966 |
| 13 | 0.1252 | 0.0668 | 0.1326 | 0.0824 | 0.1404 | 0.0936 |
| 14 | 0.1241 | 0.0643 | 0.1308 | 0.0796 | 0.1382 | 0.0908 |
| 15 | 0.1231 | 0.0620 | 0.1293 | 0.0770 | 0.1363 | 0.0883 |
| 16 | 0.1223 | 0.0599 | 0.1279 | 0.0747 | 0.1346 | 0.0858 |
| 17 | 0.1217 | 0.0580 | 0.1267 | 0.0724 | 0.1331 | 0.0836 |
| 18 | 0.1211 | 0.0564 | 0.1257 | 0.0704 | 0.1317 | 0.0815 |
| 19 | 0.1207 | 0.0549 | 0.1247 | 0.0684 | 0.1305 | 0.0794 |
| 20 | 0.1204 | 0.0536 | 0.1239 | 0.0666 | 0.1293 | 0.0775 |
| 21 | 0.1201 | 0.0524 | 0.1232 | 0.0649 | 0.1283 | 0.0757 |
| 22 | 0.1199 | 0.0514 | 0.1225 | 0.0633 | 0.1274 | 0.0740 |
| 23 | 0.1198 | 0.0505 | 0.1219 | 0.0619 | 0.1265 | 0.0724 |
| 24 | 0.1198 | 0.0498 | 0.1214 | 0.0605 | 0.1257 | 0.0709 |
| 25 | 0.1197 | 0.0491 | 0.1210 | 0.0592 | 0.1250 | 0.0694 |
| 26 | 0.1198 | 0.0485 | 0.1206 | 0.0580 | 0.1243 | 0.0680 |
| 27 | 0.1199 | 0.0480 | 0.1203 | 0.0569 | 0.1237 | 0.0667 |
| 28 | 0.1200 | 0.0475 | 0.1200 | 0.0559 | 0.1232 | 0.0654 |
| 29 | 0.1201 | 0.0471 | 0.1197 | 0.0549 | 0.1227 | 0.0642 |
| 30 | 0.1203 | 0.0467 | 0.1195 | 0.0540 | 0.1223 | 0.0630 |
